# Hypoxia‐induced miR‐3677‐3p promotes the proliferation, migration and invasion of hepatocellular carcinoma cells by suppressing SIRT5

**DOI:** 10.1111/jcmm.15503

**Published:** 2020-06-28

**Authors:** Bowen Yao, Yazhao Li, Yongshen Niu, Liang Wang, Tianxiang Chen, Cheng Guo, Qingguang Liu

**Affiliations:** ^1^ Department of Hepatobiliary Surgery The First Affiliated Hospital of Xi'an Jiaotong University Xi'an China; ^2^ Center for Translational Medicine The First Affiliated Hospital of Xi'an Jiaotong University Xi'an China

**Keywords:** hepatocellular carcinoma, hypoxia, invasion, metastasis, MicroRNA‐3677‐3p, sirtuin 5

## Abstract

Hepatocellular carcinoma (HCC), with life‐threatening malignant behaviours, often develops distant metastases and is the fourth most common primary cancer in the world, having taken millions of lives in Asian countries such as China. The novel miR‐3677‐3p is involved in a high‐expression‐related poor prognosis in HCC tissues and cell lines, indicating oncogenesis functions in vitro and in vivo. Initially, we confirmed the inhibition of proliferation, migration and invasion in miR‐3677‐3p knock‐down MHCC‐97H and SMMC‐7721 cell lines, which are well known for their high degree of invasiveness. Then, we reversed the functional experiments in the low‐miR‐3677‐3p‐expression Hep3B cell line via overexpressing miR‐3677‐3p. In nude mice xenograft and lung metastasis assays, we found suppressor behaviours, smaller nodules and low density of organ spread, after injection of cells transfected with shRNA‐miR‐3677‐3p. A combination of databases (Starbase, TargetScan and MiRgator) illustrated miR‐3677‐3p targets, and it was shown to suppress the expression of SIRT5 in a dual‐luciferase reporter system. To clarify the conclusions of previous ambiguous research, we up‐regulated SIRT5 in Hep3B cells, and rescue tests were established for confirmation that miR‐3677‐3p suppresses SIRT5 to enhance the migration and invasion of HCC. Interestingly, we discovered hypoxia‐induced miR‐3677‐3p up‐regulation benefited HCC malignancy and invasiveness. In conclusion, the overexpression of miR‐3677‐3p mediated SIRT5 inhibition, which could increase proliferation, migration and invasion of HCC in hypoxic microenvironments.

## 1 INTRODUCTION

Hepatocellular carcinoma ranks as the fourth most frequently diagnosed cancer in global surveys, and it was the third highest cause of death in China in a 2018 national health survey.^1^ With its early local and systemic metastatic features, increasing amounts of research have been focused on HCC.^2^, ^3^ Regardless of the many efforts made towards the treatment of malignancies, HCC remains incurable owing to its high metastatic potential, with most deaths from HCC being attributable to its aggressiveness and growing resistance to the existing targeted medicines.^4^, ^5^

Hypoxic environments have been confirmed in many types of cancers; they are closely related to metastasis, apoptosis and invasion,^6^, ^7^, ^8^ and therefore, identifying pathways and downstream targets of hypoxic conditions might lead to new therapeutic candidates for metastatic HCC. Angiogenesis,^9^, ^10^ reprogramming of metabolism,^11^, ^12^ extracellular matrix remodelling^13^, ^14^ and the promotion of inflationary cell chemotaxis are all triggered by hypoxia.^15^, ^16^ However, the characteristics of HCC, especially under hypoxic conditions, have not been fully understood.

A number of microRNAs (miRNAs), including miR‐21^17^, ^18^, ^19^ and miR‐221,^20^, ^21^, ^22^ have been widely studied in regard to invasive HCC. miR‐876^23^ and miR‐1468^24^ along with others have been reported in various articles. Therefore, there is great potential for identifying an underlying miRNA that can be used to develop treatments for HCC and assist in early detection of HCC and the control of metastasis.^25^, ^26^, ^27^

miRNAs play an independent role in the pathogenesis and progression of almost all types of cancers, such as non‐small cell lung cancer,^28^, ^29^ breast cancer,^30^, ^31^, ^32^ gastric carcinoma^33^, ^34^, ^35^ and bladder tumours.^36^, ^37^ They are responsible for modulation of their cellular differentiation, as well as in regulating the transcription of their downstream targets.

A novel miRNA, miR‐3677‐3p, has never been reported in any article about neoplasms, including HCC. With the support of advanced analysis of a considerable amount of HCC samples, we identified miR‐3677‐3p and investigated its function in HCC and in the hypoxia‐regulated axis in HCC.

Sirtuin 5 (SIRT5) was initially discovered in 1999, and the five human sirtuins, SIRT1 to SIRT5, are widely expressed in foetal and adult tissues.^38^ The various members of the sirtuin family have different cellular locations, with SIRT1, SIRT6 and SIRT7 in the nucleus, while SIRT3, SIRT4 and SIRT5 are localized in mitochondria, and some researchers have compared traditional histone deacetylases (HDACs) to sirtuins.^39^ All members of this family play similar but different roles in gene expression and molecular modifications. Previous studies have claimed that SIRT5 differential expression could lead to suppressed cellular growth, endoplasmic reticulum stress (ER stress), senility and apoptosis in various cancers.^40^, ^41^, ^42^, ^43^, ^44^, ^45^, ^46^

Although the research into the function of SIRT5 in HCC just began in 2018,^47^ several experiments have shown multiple roles of SIRT5 in HCC. However, there has not yet been any final verdict reached on whether SIRT5 has a positive or negative role in HCC, although it is expressed at only low levels in tumour specimens.^41^, ^46^, ^48^, ^49^ Thus, we investigated its phenomenon of restraint in functional and rescue experiments.

Therefore, in this study, we evaluated the potential mechanism of the metastatic and invasive promoter, miR‐3677‐3p, in HCC and found that it targeted SIRT5 in hypoxia conditions.

## 2 MATERIALS AND METHODS

### 2.1 Patients and tissue samples

Hepatocellular carcinoma tissues and adjacent tissues were obtained from 137 patients in the First Affiliated Hospital of Xi'an Jiaotong University from January 2013 to December 2014 for the current study. This study was approved by the Ethics Committee of the First Affiliated Hospital of Xi'an Jiaotong University, and signed informed consent was obtained from all participants prior to the study. The patients had a mean age of 62.4 ± 8.6 years, including 65 patients under the age of 65 years and 72 patients older than 65. A total of 114 male patients and 23 female patients were included in this study. According to Edmondson and the tumour‐node‐metastasis stages, 89 and 87 cases were in early stages and 48 and 50 cases were in advanced stages, respectively. In addition, tumour number, tumour size in centimetres, venous invasion, hepatitis B virus surface antigen (HBVsAg) and alpha‐fetoprotein (AFP) were collected and analysed by statistical methods.

Some indispensable inclusion criteria when choosing suitable patients for our further surveys were as follows: (a) a pathological diagnosis of HCC had been made; (b) no previous treatments involving radiotherapy, chemotherapy or immunotherapy had been administered prior to surgery; (c) samples for research only came from patients who had undergone liver resection for HCC; (d) complete clinical and pathological information as well as the patient's outcome were available.

### 2.2 Cell Counting Kit‐8 (CCK‐8)

A CCK‐8 kit (#CK04‐05, Dojindo Molecular Technologies Maryland, USA) was used for the determination of cell viability. The cells were seeded in 96‐well plates at a density of 3500 cells per well in 10% foetal bovine serum (FBS) Dulbecco's modified Eagle medium (DMEM) for 0, 24, 48 or 72 hours. The total cell numbers were assessed after incubation with 10 μL of CCK8 for another 0.5 hours. The optical density (OD) measured by a microplate reader at 450 nm was reported as the means ± SD. The experiments were repeated three times.

### 2.3 Haematoxylin and eosin (H&E) staining

The whole staining process consists of five steps: dewaxing, dyeing, dehydration, transparency and sealing. After dewaxing, we stained the slides in haematoxylin for 10 minutes.^50^ Then, after washing away the haematoxylin and allowing the colour to develop for another 2 minutes, we placed the slide for 10 seconds in 1% hydrochloric acid alcohol, so the colour faded to red. Next, we washed the slides for 2 minutes in water and put them into the red eosin dye for 4 minutes. The slides were rinsed in water, dehydrated and sealed with a coverslip.

### 2.4 Dual‐luciferase reporter gene assay

A dual‐luciferase reporter gene assay was established to demonstrate that miR‐3677‐3p targeted SIRT5. The 3′‐untranslated region (3′UTR) of the SIRT5 gene was cloned into mi‐vector (#E1330, Promega, Madison, WI, USA). Site‐directed mutagenesis was performed on the potential binding sites of miR‐3677‐3p, and the SIRT5‐mutant (MUT) vector or SIRT5‐wild (WT) was also constructed. The RL‐vector containing Renilla luciferase (#E2241, Promega, WI, USA) was used as the internal control. The miR‐3677‐3p mimics or the NC plasmids were transfected into MHCC‐97H cells together with SIRT5‐MUT or SIRT5‐WT. Two days after transfection, luciferase activity was detected in the cells according to the protocol of the luciferase assay kit (GeneCopoeia, Rockville, MD, USA).

### 2.5 Cell culture

Hepatocellular carcinoma cell lines MHCC‐97H, Hep3B and SMMC‐7721 (Shanghai Institute of Biochemistry and Cell Biology Chinese Academy of Sciences, Shanghai, China) were incubated in DMEM (HyClone, GE, Buckinghamshire, England) supplemented with 10% FBS (Gibco, Thermo Fisher, Shanghai, China) and incubated in 5% CO2 at 37°C. The cDNA from other HCC cell lines like Huh7, HepG2 and MHCC‐97L was tested. Cells were classified into several groups after different treatments. We had negative controls (NC) (6 groups including HCC cells transfected with miR‐3677‐3p inhibitor NC in Figures 2,5 and 8, miR‐3677‐3p NC plasmids in Figures 3 and 5, sh‐miR‐NC in Figure 4, SIRT5 NC in Figure 6 and miR‐3677‐3p inhibitor NC/miR‐3677‐3p NC + SIRT5 si‐NC in Figure 7), miR‐3677‐3p mimics (Hep3B transfected with miR‐3677‐3p mimics plasmid in Figure 5), miR‐3677‐3p inhibitor (MHCC‐97H and SMMC‐7721 cells transfected with miR‐3677‐3p inhibitor), SIRT5 siRNA1 in MHCC‐97H cells and miR‐3677‐3p inhibitor + SIRT5 siRNA1 (MHCC‐97H cells transfected with miR‐3677‐3p inhibitor + SIRT5 siRNA1). The transfections were performed according to the instructions of the manufacturer of Lipofectamine 2000 (#11668019, Invitrogen, CA, USA).

### 2.6 Reverse transcription quantitative polymerase chain reaction (RT‐qPCR)

Levels of SIRT5, HIF‐1a, miR‐3677‐3p and beta‐actin were determined using RT‐qPCR. The total RNA was extracted with TRIzol (Invitrogen Inc, Carlsbad, CA, USA) following the manufacturer's instructions, and then the RNA was reverse transcribed into cDNA (#k1622, Thermo Fisher, Shanghai, China). The PCR program was performed following the kit's protocol (for reverse transcription, 42°C for 5 minutes, 70°C for 62 minutes and 4°C at standby; for qPCR, 95°C for 10 minutes, 95°C for 5 seconds for cDNA or 95°C for 15 seconds for the miRNA RT product, 60°C for 30 seconds and 72°C for 30 seconds for 40 cycles within the last 3 steps). Beta‐actin and U6 were used as internal references for mRNA and miRNA, respectively. The fold changes were calculated by means of relative quantification (2^−ΔΔCT^ method).

### 2.7 Western blotting

A bicinchoninic acid (BCA) Protein Assay Kit (#WB003, FiveHeart, Xi'an, China) was used for measurement of the protein concentration after the total protein had been extracted in RIPA Lysis Buffer (#WB009, Five Heart, Xi'an, China). After separation with 8% (for HIF‐1a) and 12% (for the other proteins) sodium dodecyl sulphate‐polyacrylamide gel electrophoresis (SDS‐PAGE), the proteins were transferred onto polyvinylidene fluoride membranes (PVDF, #IPVH00010, Merck‐Millipore, , Darmstadt, Germany). The membranes were blocked with 10% skimmed milk and then incubated at 4°C for 12 hours with the primary antibodies: mouse monoclonal antibody against β‐actin (#ab6276, 1:5000, Abcam, Cambridge, UK), rabbit polyclonal antibody against SIRT5 (#8782, Cell Signaling Technology, Danvers, MA, USA) and rabbit monoclonal antibody against HIF‐1a (#ab16066, 1:2500, Abcam). After washing three times with Tris‐buffered saline supplemented with 0.05% Tween 20 (TBST), the membrane was incubated with goat anti‐mouse IgG secondary antibody (#ab7072, 1:6000, Abcam) or goat anti‐rabbit IgG secondary antibody (#ab6721, 1:6000, Abcam Inc) at room temperature for one and a half hours. Next, the membrane was rinsed three times with PBST and exposed to western HRP substrate (#WBLUF0110, Merck‐Millipore, Darmstadt, Germany) under dark conditions. Relative protein levels of the target genes were expressed as the ratio of grey values of the target protein bands to those of the β‐actin band.

### 2.8 5‐ethynyl‐2‐deoxyuridine (EDU) assay

DNA replication was tested by using an EDU Labeling/Detection Kit (#R11053.9, Ribobio, Guangzhou, China). Cells grown in 24‐well plates at a density of 12 × 10^5^ cells/well were incubated with 325 μL EDU (50 μmol/L) labelling media overnight at 37°C. After treatment with 4% paraformaldehyde and washing with 0.5% Triton X‐100 and glycine/PBS several times, the plates were stained with Apollo working solution. Finally, DAPI was used to stain the cell nuclei. The proportion of EDU‐positive cells was calculated after fluorescence microscopic analyses.

### 2.9 Transwell assays

The 8‐μm Transwell chambers (Corning, Acton, MA, USA) placed in 24‐well plates were used for migration and invasion assays. In the invasion assays, the chamber was coated with a 70 μL mixture of Matrigel (#356234, BD, California, SA, USA) and DMEM at a 1:6 ratio. Then, 200 μL of MHCC‐97H or SMMC‐7721 transfected cells were seeded into the apical chambers, with complete medium added into the lower chamber. The chambers were then incubated at 37°C with 5% CO_2_. After a 36 hours incubation, the cells were fixed and stained with 0.1%‐0.5% crystal violet. For the migration assay, the same procedure was followed omitting the Matrigel. The stained cells were counted under the microscope to determine the average number of cells in five randomly selected fields.

### 2.10 Tumour formation and lung metastasis model in nude mice

An MHCC‐97H cell suspension was prepared after digestion by trypsin‐EDTA (HyClone, GE, Buckinghamshire, England). A mixture of PBS and Matrigel (#356234, BD, California, USA) at a ratio of 1:1 was prepared for suspending cells transfected with sh‐NC and sh‐miR‐3677‐3p at a final concentration of 3 × 10^6^ cells/150 μL.

A total of 16 male 4‐week‐old nude mice (SLAC Laboratory Animal Co. Ltd., Shanghai, China) were divided into two groups, a xenograft group and a lung metastasis group, with eight mice per group. The mice were allowed to adapt for one week, and then they were anaesthetized with chloral hydrate.

The xenograft group was inoculated subcutaneously in the left posterior cervical subcutaneous site with the prepared cells. The mice were kept in the same specific pathogen‐free environment and were observed and measured weekly after the injections. Tumour length and width were recorded, and the gross tumour volume was calculated according to the international common formula, volume = (length × width^2^)/2. On the 28th day, the mice were sacrificed by chloral hydrate and the tumours were removed and weight.

For the lung metastasis model, the same procedure was followed except the cells were injected slowly into the tail vein at 3 × 10^6^ cells/150 μL. The mice were euthanized after 42 days, and pneumonectomy was performed. The number of lung metastasis nodules was counted under a 100‐fold high‐power field of a microscope on sections after H&E staining. All animal experiments were approved by the Institutional Animal Care and Use Committee of the Xi'an Jiaotong University, Xi'an, China.

### 2.11 Statistical analysis

The statistical analysis was conducted by using SPSS 19.0 (IBM, Armonk, USA) and GraphPad 6.0 (GraphPad Software, San Diego, USA). Mean ± standard deviation was used for measurement data. Two groups’ data were compared using t tests, and multiple‐groups data were compared using one‐way analysis of variance (ANOVA). Fischer's least significant difference (LSD) method was adopted for paired comparisons. Enumeration data are presented as percentages and were analysed using chi‐square tests. Values of *P* < 0.05, *P* < 0.01 or *P* < 0.001 were defined as varying levels of statistical significance.

## 3 RESULTS

### 3.1 HCC presenting with high expression of miR‐3677‐3p had a poor prognosis

**FIGURE 1:**
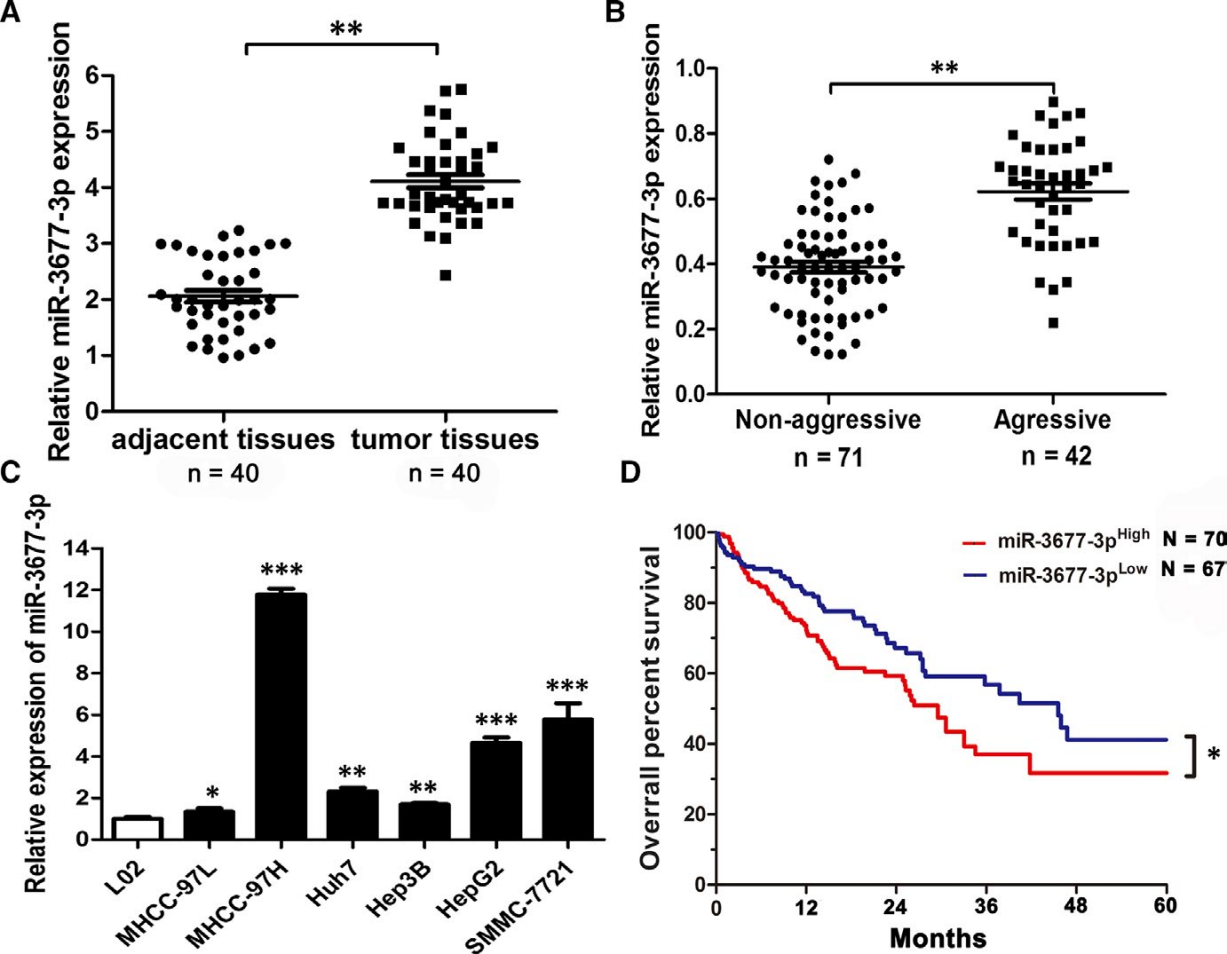
HCC with high expression of miR‐3677‐3p has a poor prognosis Comparing differences in the expression levels of miR‐3677‐3p between (A) HCC and matched nontumour tissues; (B) aggressive and non‐aggressive tumour tissues; and (C) HCC cell lines and the immortalized hepatic cell line LO2, U6 was used as internal control. D, Overall survival was compared between HCC patients with high‐expression levels of miR‐3677‐3p and those with low levels of miR‐3677‐3p. *P < .05, **P < .01, ***P < .001

According to databases analyses, we found many miRNAs with abnormal expression in deceased or living samples (OncomiR: http://www.oncomir.org/). In HCC tissues, miR‐3677‐3p was expressed at a high level and it was correlated with a poor prognosis. Its significant up‐regulation in 374 tumour samples was obvious (Figure S1A,B from Starbase: http://starbase.sysu.edu.cn/ and OncomiR). Then, we tested its expression in liver cancer specimens from our medical institution, which consisted of 40 pairs of adjacent normal and liver tumour tissues, and 42 invasive and 71 non‐aggressive tumours. The expression of miR‐3677‐3p was increased in the tumours relative to the normal tissue, and it was also increased in the aggressive tumours relative to the non‐aggressive tumours as expected (Figure 1A,B). Similarly, the results of relative expression in HCC cell lines indicated that the MHCC‐97H and SMMC‐7721 cell lines, well known for being invasive, expressed miR‐3677‐3p at high levels. miR‐3677‐3p was expressed at lower levels in noninvasive cell lines like Hep3B, although even in noninvasive HCC cell lines, its expression level was remarkably elevated relative to normal hepatic cells (Figure 1C).

Considering the poor prognostic features of HCC, we investigated the correlation between the prognosis and the expression level of this miRNA. We found higher expression of miR‐3677‐3p was correlated with a low survival rate (Figure 1D). In addition, the TCGA database (OncoLnc: http://www.oncolnc.org/) corroborated the correlation between miR‐3677‐3p expression, using the median as the cut‐off, with a lower survival percentage over 10 years (Figure S1C).

### 3.2 Clinical significance of miR‐3677‐3p in HCC patients

To further confirm that expression of miR‐3677‐3p was associated with the clinical features of HCC patients, we divided 160 HCC patients into high and low groups according to the median value of miR‐3677‐3p expression. Although 13 and 10 patients’ clinical information were incomplete, either blood test results missing or multiple other treatments were applied before hepatectomy, we still had 67 cases in the low‐expression group and 70 in the high‐expression group for subsequent research. As shown in Table 1, we demonstrated that miR‐3677‐3p up‐regulation was significantly associated with tumour‐node‐metastasis (TNM) stage (III + IV), Edmondson stage (III + IV), venous invasion and multiple tumour nodes. These data suggest that miR‐3677‐3p is a potential biomarker for the clinical outcome of HCC patients. To test the clinical values and prognostic independent risk factors in our study, univariate and multivariate Cox hazard analysis was applied for making confirmation that miR‐3677‐3p was one of the independent risk factor for survival of HCC patients (Table 2).

**TABLE 1:** Correlation between the clinicopathologic characteristics and miR‐3677‐3p expression in HCC (n = 137)

Clinical parameters	Cases	Expression level		*P* value
		MiR‐3677‐3p^high^ (n = 70)	MiR‐3677‐3p^low^ (n = 67)	
Age (years)				
<65 y	72	35	37	.608
≥65 y	65	35	30	
Gender				
Male	114	62	52	.110
Female	23	8	15	
Tumour size (cm)				
<5 cm	50	27	23	.723
≥5 cm	87	43	44	
Tumour number				
Solitary	80	28	52	**.001**
Multiple	57	42	15	
Edmondson				
I + II	89	32	57	**.003**
III + IV	48	38	10	
TNM stage				
I + II	87	31	56	**.011**
III + IV	50	39	11	
Venous invasion				
Present	73	29	44	**.016**
Absent	64	41	23	
AFP				
<400 ng/mL	61	35	26	.229
≥400 ng/mL	76	35	41	
HBVsAg				
Positive	114	56	58	.364
Negative	23	14	9	

Note

Bold font statistically significant.

Abbreviations: AFP, alpha‐feto protein; HCC, hepatocellular carcinoma; TNM, tumour‐node‐metastasis.

**TABLE 2:** Univariate and multivariate cox hazard analysis of clinical features for survival

	Univariate analysis		Multivariate analysis	
	HR	95% CI	HR	95% CI
Age	1.02	0.877‐1.135	—	—
Gender	0.785	0.674‐1.022	—	—
Tumour size	2.233	2.036‐2.523	—	—
Tumour number	**2.537**	**2.327‐1.876**	**2.353**	**2.214‐2.581**
Edmondson stage	**2.582**	**1.864‐2.933**	**2.128**	**1.826‐2.759**
TNM stage	**2.351**	**2.015‐2.574**	**2.305**	**2.134‐2.704**
Venous invasion	**2.106**	**1.772‐2.486**	**1.986**	**1.688‐2.233**
AFP	1.163	0.876‐1.355	—	—
HBsAg	1.055	0.714‐1.701	—	—
miR‐3677‐3p	**2.252**	**2.103‐2.421**	**2.222**	**2.072‐2.393**

Note

Bold font statistically significant.

Abbreviations: CI, confidence interval; HR, Hazard ratio.

### 3.3 miR‐3677‐3p promotes metastasis, invasion and proliferation of HCC

**FIGURE 2:**
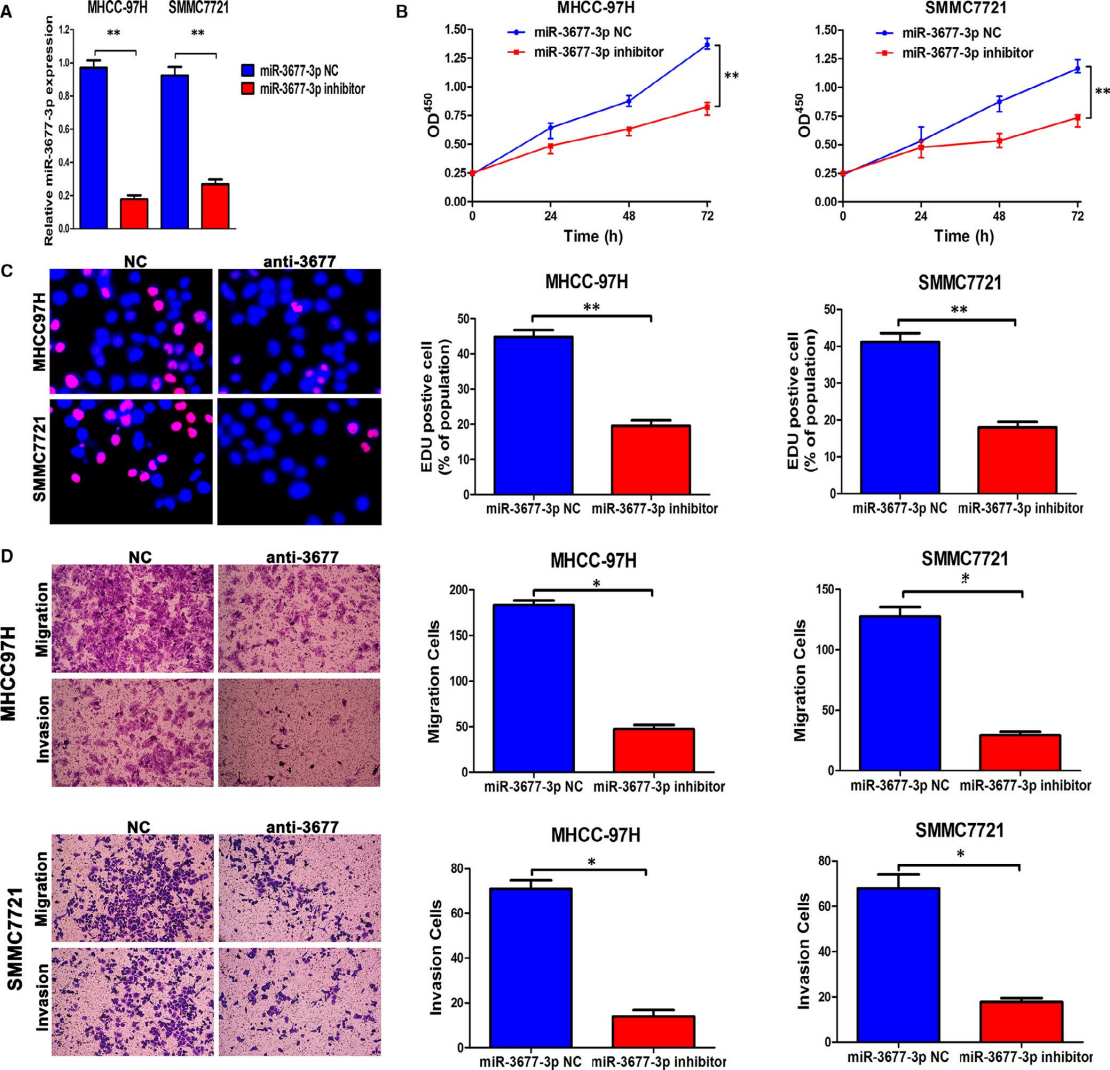
Down‐regulated miR‐3677‐3p inhibits metastasis, invasion and proliferation of HCC in vitro. A, MHCC‐97H and SMMC‐7721 cells that were transfected with the corresponding miRNA inhibitor were subjected to qRT‐PCR for evaluation of miR‐3677‐3p expression. B, CCK‐8 tests showed lower cellular vitality in both cell lines transfected with miR‐3677‐3p inhibitor. C, The proportion of EDU in two cell lines transfected with miR‐3677‐3p inhibitor was lower than in the NC group. Bar figures indicate the statistics. D, Cell migration and invasion as measured by Transwell assays were inhibited by knock‐down of miR‐3677‐3p in MHCC‐97H and SMMC‐7721 cells. Bar figures indicate the statistics. n = 4 independent experiments. *P < .05, **P < .01

Functional experiments were established to evaluate miR‐3677‐3p in HCC cell lines. MHCC‐97H and SMMC‐7721, both with high expression of miR‐3677‐3p, were selected as cell lines for its knock‐down (Figure 1C). First, real‐time PCR was applied to evaluate the knock‐down efficiency (Figure 2A).

Compared with the negative control groups of the two cell lines, the CCK‐8 assay indicated that the vitality of the HCC cells was diminished after miR‐3677‐3p knock‐down. Similarly, complementary EDU assays illustrated proliferation suppression in both cell lines (Figure 2B,C).

We next evaluated the important behaviours, migration and invasion, in Transwells with or without Matrigel. The invasive and migratory characteristics of MHCC‐97H and SMMC‐7721 were both inhibited after miR‐3677‐3p knock‐down (Figure 2D).

**FIGURE 3:**
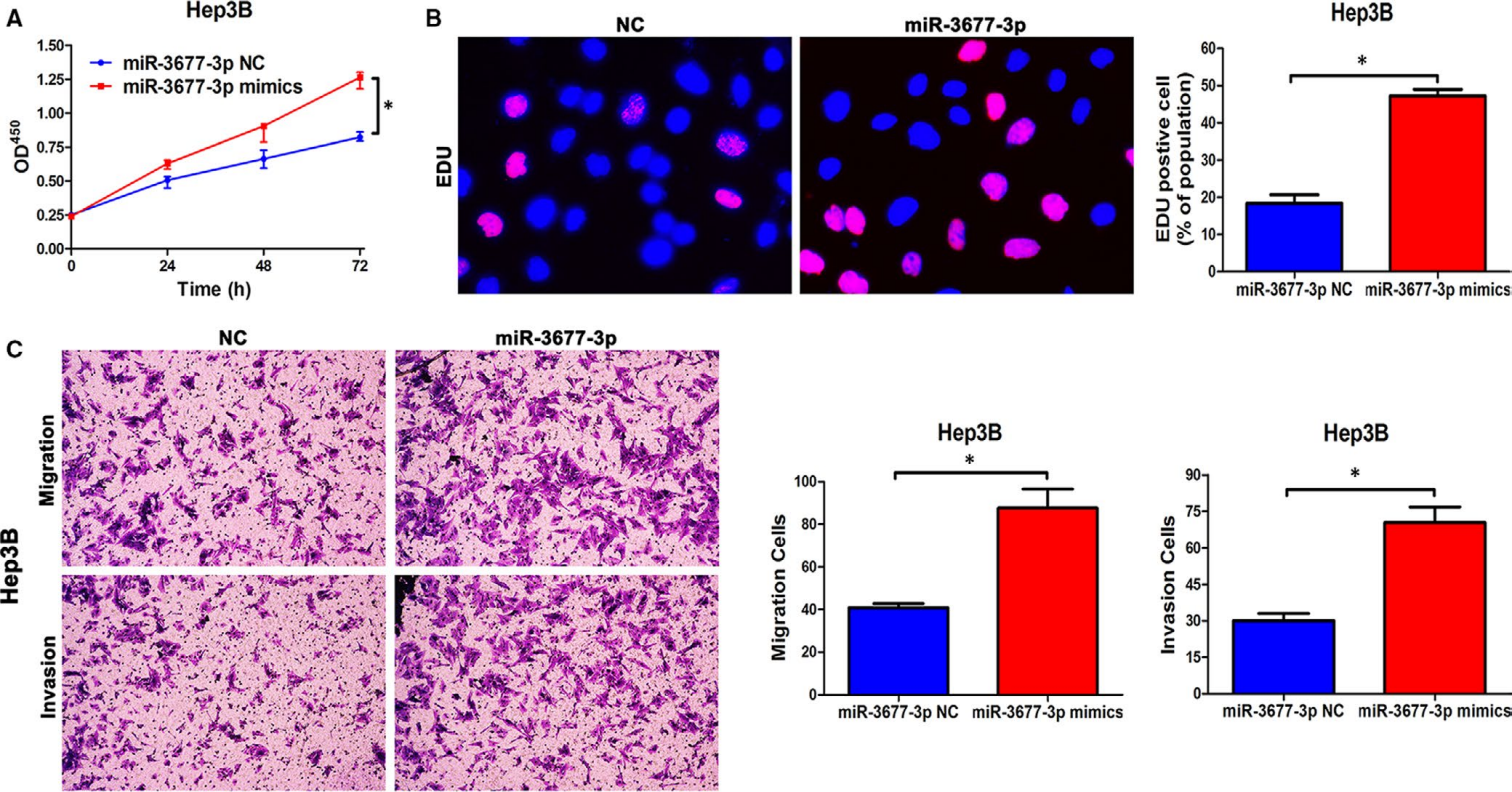
Up‐regulated miR‐3677‐3p promotes metastasis, invasion and proliferation of HCC in vitro. A, CCK‐8 tests showed higher cellular vitality in cell lines transfected with miR‐3677‐3p overexpression plasmid. B, The proportion of EDU in Hep3B cells transfected with miR‐3677‐3p overexpression plasmid was higher than in the NC group. Bar figures indicate the statistics. C, Cell migration and invasion as measured by Transwell assays were enhanced by up‐regulation of miR‐3677‐3p in Hep3B cells. Bar figures indicate the statistics. n = 4 independent experiments. *P < .05

Next, overexpression of miR‐3677‐3p, tested by qPCR, was used to support the results that miR‐3677‐3p promotes cell growth, metastasis and invasion (Figure S2A). The Hep3B cell line was chosen for these experiments, and we found miR‐3677‐3p did indeed enhance proliferation, invasive and migration (Figure 3).

### 3.4 Knocking‐down miR‐3677‐3p blocked the growth of xenografts and lung metastasis in nude mice

**FIGURE 4:**
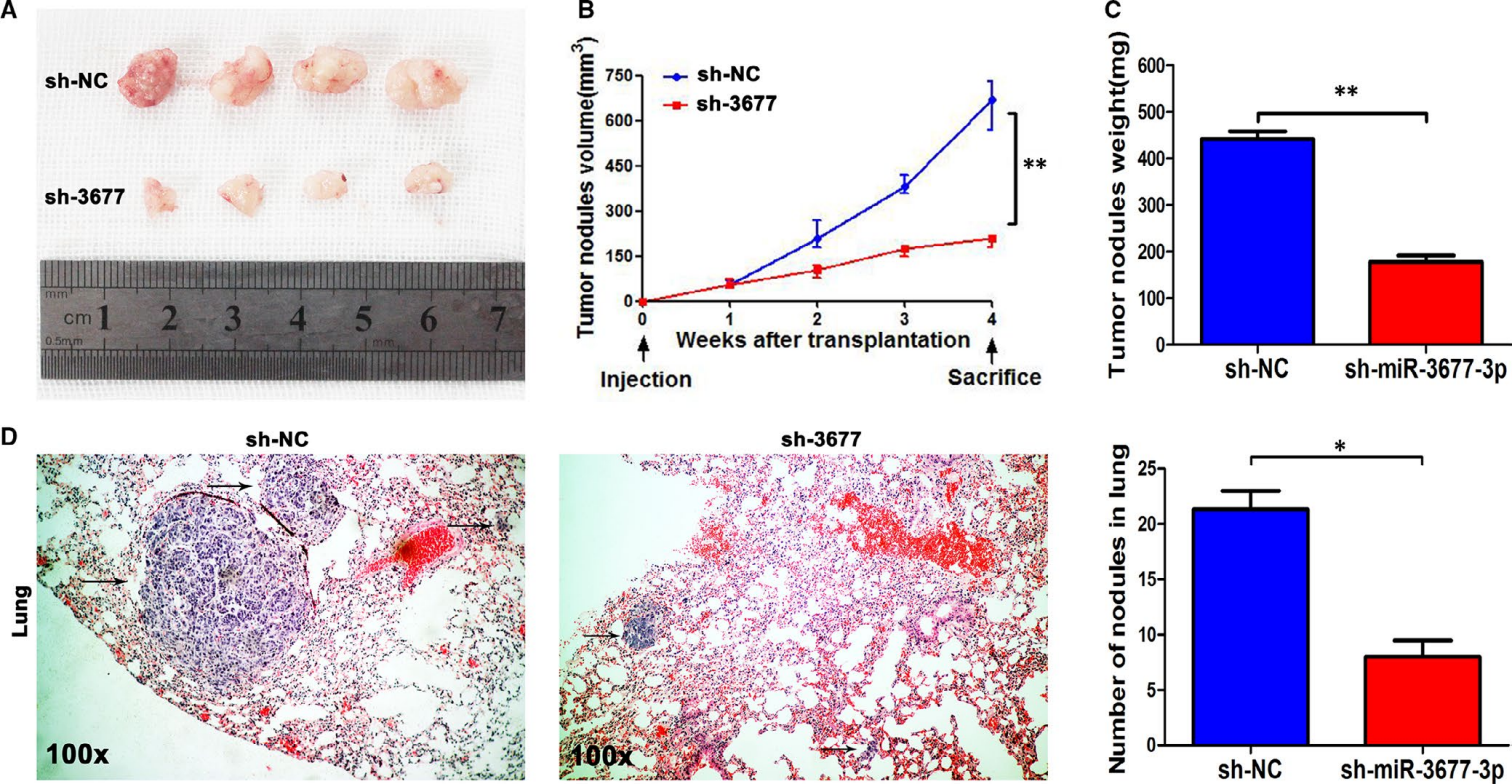
miR‐3677‐3p inhibits the growth of nodules and lung metastasis of MHCC‐97H cells in nude mice. A, Nodules comparison figure for the Sh‐miR‐3677‐3p and sh‐NC groups. B, Week‐measurement curves of nodules showing smaller volumes in the sh‐miR‐3677‐3p group. C, Tumour weight of the subcutaneous nodules. (D) Representative HE staining of lung metastases of the Sh‐miR‐3677‐3p and sh‐NC cells. Bar figures indicate the statistics of the number of lung nodules. Arrows mark the HCC nodules. *P < .05, **P < .01

To confirm the tumour‐promoting effects of miR‐3677‐3p in vivo, we used tumour xenograft models. The group with miR‐3677‐3p showed decreased expression, verified by post‐transfection sh‐miR‐3677‐3p qPCR, illustrated a weakened capacity of the HCC xenografts (Figure S2B and Figure 4A). Measurement of the injected tumour nodules suggested significant suppression of tumour volume in the sh‐miR‐3677‐3p group (Figure 4B). Not only the weight of the nodules but also the weight of the mice themselves confirmed that the tumour sites in the negative group were larger than the sh‐group and the mice had no statistical differences in regards to weight, sex or age (Figure 4C and Figure S2C). Models of lateral vein injection were used, and they showed fewer and smaller foci in the lungs of nude mice via microscopic evaluation (Figure 4D). Taken together, these results suggest that miR‐3677‐3p promotes oncogenesis and metastatic behaviours of HCC in vivo.

### 3.5 SIRT5 is a target of miR‐3677‐3p

**FIGURE 5:**
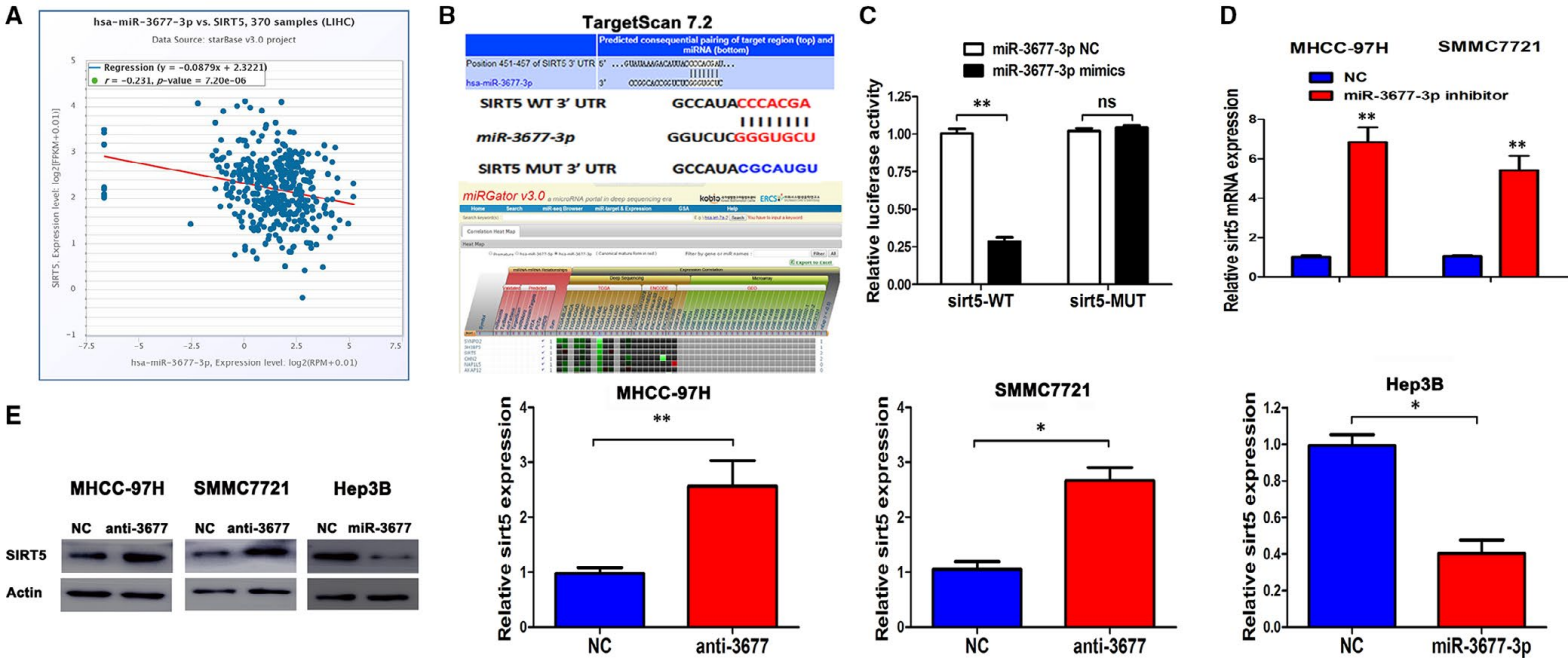
SIRT5 is a direct target of miR‐3677‐3p in HCC cells. A, Relationship of miR‐3677‐3p and SIRT5 in HCC in the Starbase database. B, Targetscan showing an miR‐3677‐3p putative binding sequence in the 3’‐UTR of SIRT5 and MiRgator supports the strong negative correlation in HCC. A mutant binding site was generated in the complementary site for the seed region of miR‐3677‐3p. C, miR‐3677‐3p significantly suppresses the luciferase activity of the wild type (WT) but not mutant (MT) 3'‐UTR of SIRT5, which led to a notable increase in the luciferase activity of the wt 3'‐UTR of SIRT5. D, qRT‐PCR analysis of SIRT5 mRNA expression in cells with miR‐3677‐3p inhibitor or NC transfection in MHCC‐97H and SMMC‐7721 cells. n = 4 repeats with similar results. E, Overexpression or down‐regulation of miR‐3677‐3p reduced or increased the expression of SIRT5 protein in Hep3B or MHCC‐97H/SMMC‐7721 cells, respectively. Bar figures indicate the statistics. *P < .05

Since SIRT5 was predicted to be a target of miR‐3677‐3p by three databases including Starbase v3.0, TargetScan v7.2 (http://www.targetscan.org/vert_72/) and MiRgator v3.0 (http://mirgator.kobic.re.kr/) (Figure 5A,B), a dual‐luciferase reporter gene assay was performed to verify the relationship between miR3677‐3p and SIRT5. Compared with the miR‐NC group, there was a clear decrease in the luciferase activity of WT‐SIRT5 in the miR‐3677‐3p mimic transfected group (Figure 5C). In addition, there was no statistically significant difference in the luciferase activity of the MUT‐SIRT5 group. In regard to the mRNA and protein level of SIRT5, there were converse changes in response to over‐ or underexpression of miR‐3677‐3p (Figure 5D,E). Therefore, miR‐3677‐5p could specifically bind to SIRT5 gene.

### 3.6 SIRT5 plays an inhibitory role in HCC

As indicated previously, research into the function of SIRT5 in HCC began in 2018 and several studies have shown multiple roles of SIRT5 in HCC. However, there has been no final verdict as to whether SIRT5 has a positive or negative role in HCC, although it has a relatively low‐expression level in tumour specimens in different databases (data not shown). qPCR testing of SIRT5 mRNA expression in HCC cell lines and the Hep3B cell line led us to select a cell line with a median level of expression of SIRT5 to conduct additional experiments (Figure S2D).

**FIGURE 6:**
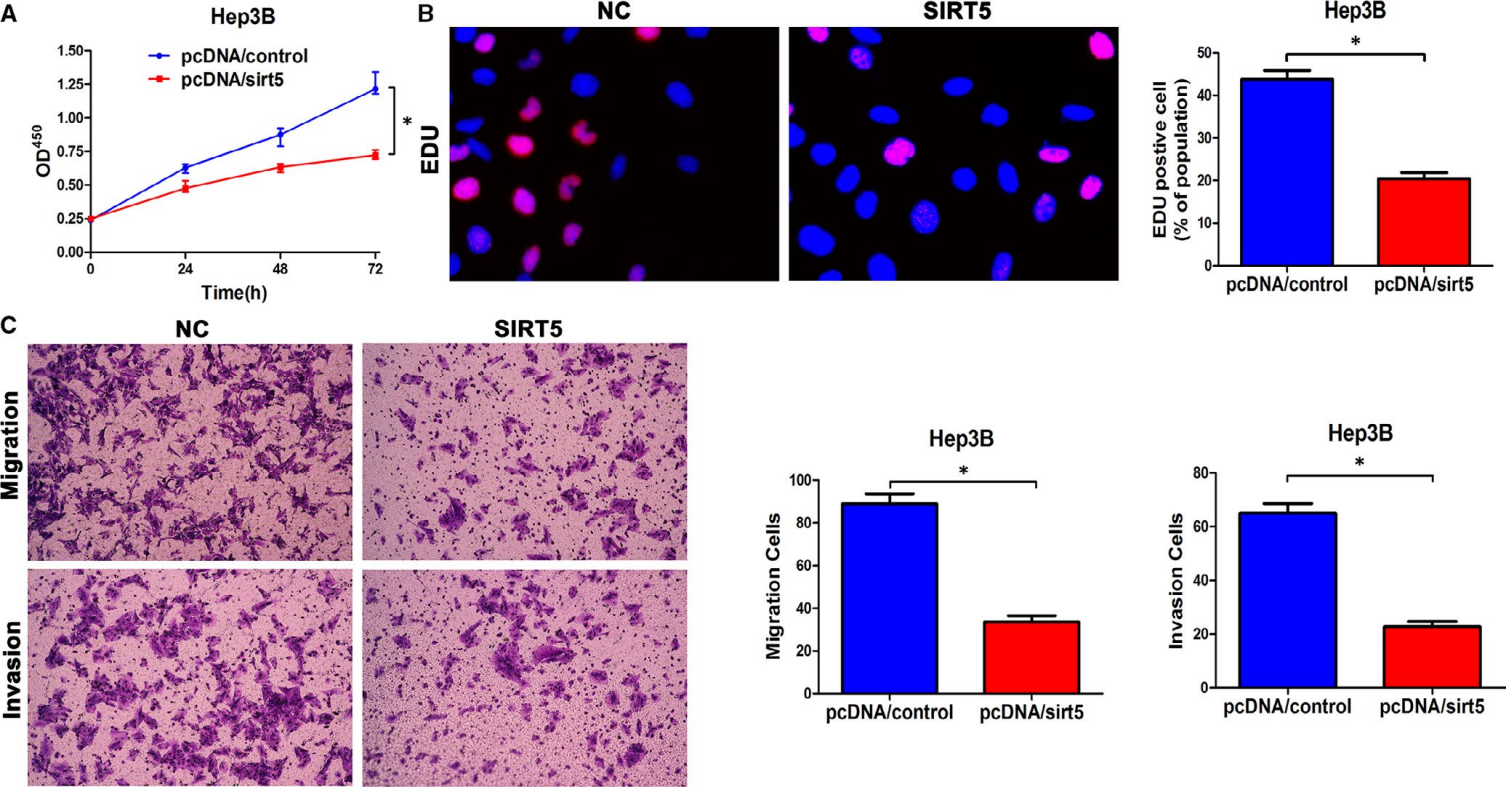
SIRT5 is down‐regulated in HCC and regulates the migration and invasion of HCC. A, CCK‐8 tests showing a lower cellular vitality in Hep3B cell lines transfected with pcDNA‐SIRT5. B, The proportion of EDU in Hep3B cells transfected with pcDNA‐SIRT5 was lower than in the NC group. Bar figures indicate the statistics. C, Cell migration and invasion as measured by Transwell assays were inhibited by up‐regulated expression of SIRT5 in Hep3B cells. Bar figures indicate the statistics. n = 4 independent experiments. *P < .05

We overexpressed SIRT5 in HCC cells (Figure S2E). CCK‐8, EDU, and Transwell invasion and migratory assays showed it had an inhibitory effect (Figure 6). To investigate the SIRT5 expression in HCC samples, we chose to measure the expression of mRNA level in samples by qPCR, then we found that SIRT5 showed a lower level in HCC (Figure S4A). In vivo, we summarized the data of tumour transplantation nodules volume, weight and calculation of lung metastasis sites in HPF of H&E. All the data in vivo illustrated the inhibition of tumour growth and lung metastasis via overexpressed SIRT5 (Figure S4B,C,D). In short, SIRT5 plays the role of an inhibitor in liver cancer.

### 3.7 Up‐regulation of miR‐3677‐3p or silencing of SIRT5 enhances HCC cell proliferation, metastasis and invasion

**FIGURE 7:**
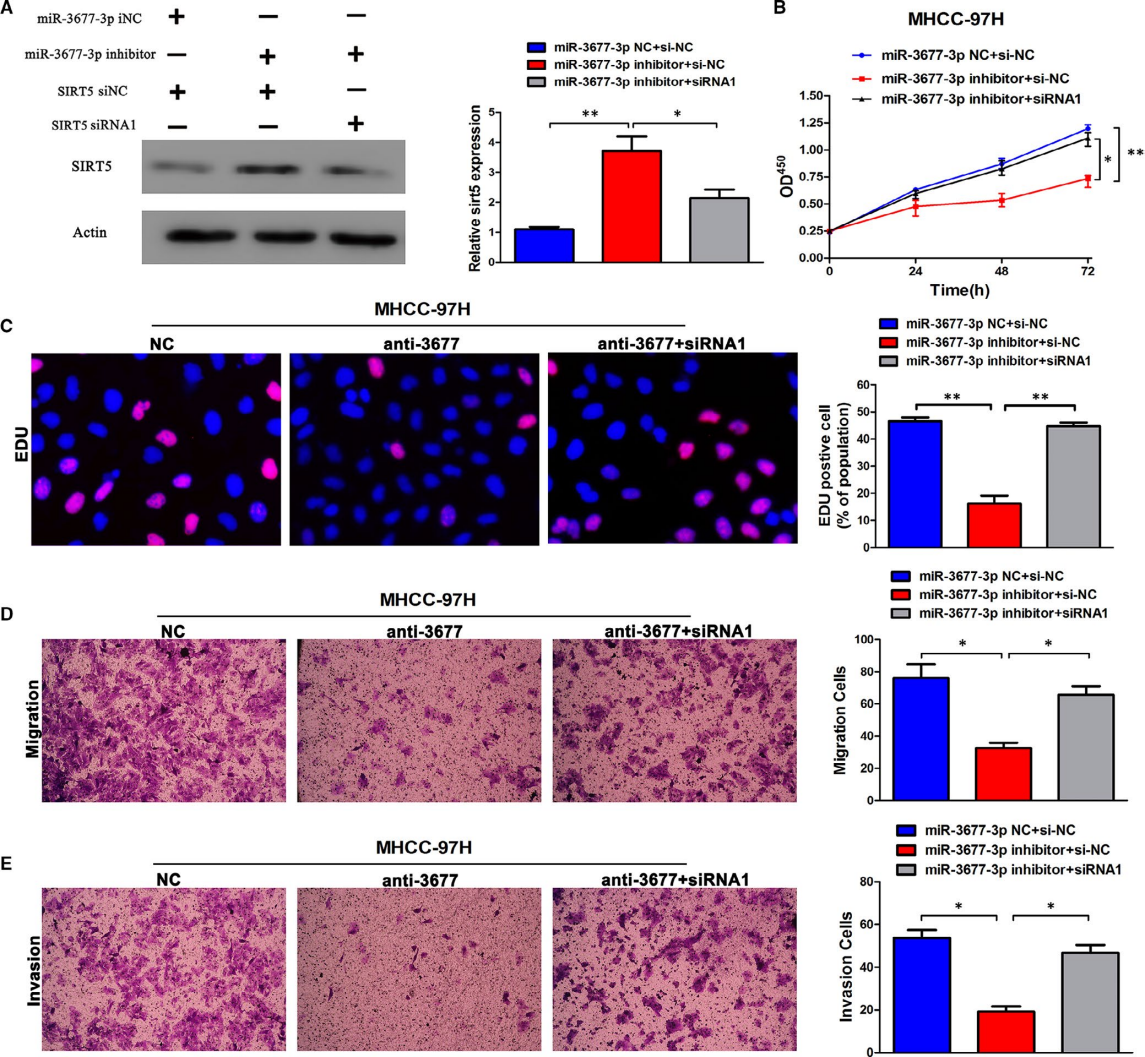
Alterations of SIRT5 partially abolish miR‐3677‐3p‐mediated HCC cell proliferation, migration and invasion. A, miR‐3677‐3p‐underexpressing MHCC‐97H cells that were transfected with inhibitor or SIRT5 expression siRNA were subjected to Western blot analysis for SIRT5. B, CCK‐8 tests showing lower cellular vitality in both cell lines transfected with miR‐3677‐3p inhibitor. SIRT5 knock‐down abrogated the effects of miR‐3677‐3p knock‐down in MHCC‐97H cells. C, The proportion of EDU in MHCC‐97H cells transfected with miR‐3677‐3p inhibitor was lower than in the NC group. SIRT5 knock‐down abrogated the effects of miR‐3677‐3p knock‐down in MHCC‐97H cells. Bar figures indicate the statistics. D, Cell migration and invasion as measured by Transwell assays were inhibited by knock‐down of miR‐3677‐3p in MHCC‐97H cells. SIRT5 knock‐down abrogated the effects of miR‐3677‐3p knock‐down in MHCC‐97H cells. Bar figures indicate the statistics. n = 4 independent experiments. *P < .05, **P < .01

Next, compared with the NC groups, a significant decrease in SIRT5 protein level with miR‐3677‐3p inhibitor and rescue with miR‐3677‐3p inhibitor + si‐SIRT5 groups (Figure 7A) was tested, while the siRNA efficiency was tested primarily in the MHCC‐97H cell line (Figure S2F). Moreover, when compared with the NC groups, the cell vitality measured by EDU and CCK‐8 in the anti‐miR‐3677‐3p group was diminished, and at the same time, there was a significant difference detected in the miR‐3677‐3p inhibitor + si‐SIRT5‐1 group (Figure 7B,C). The same trend was seen in Transwell assays, and the influence of anti‐miR‐3677‐3p and silencing SIRT5 simultaneously increased the aggressive nature of the MHCC‐97H cell line (Figure 7D,E). These findings suggest that miR‐3677‐3p can promote the carcinogenesis level of HCC by inhibiting the expression level of SIRT5.

### 3.8 Hypoxia induces the promotion of miR‐3677‐3p carcinogenesis

**FIGURE 8:**
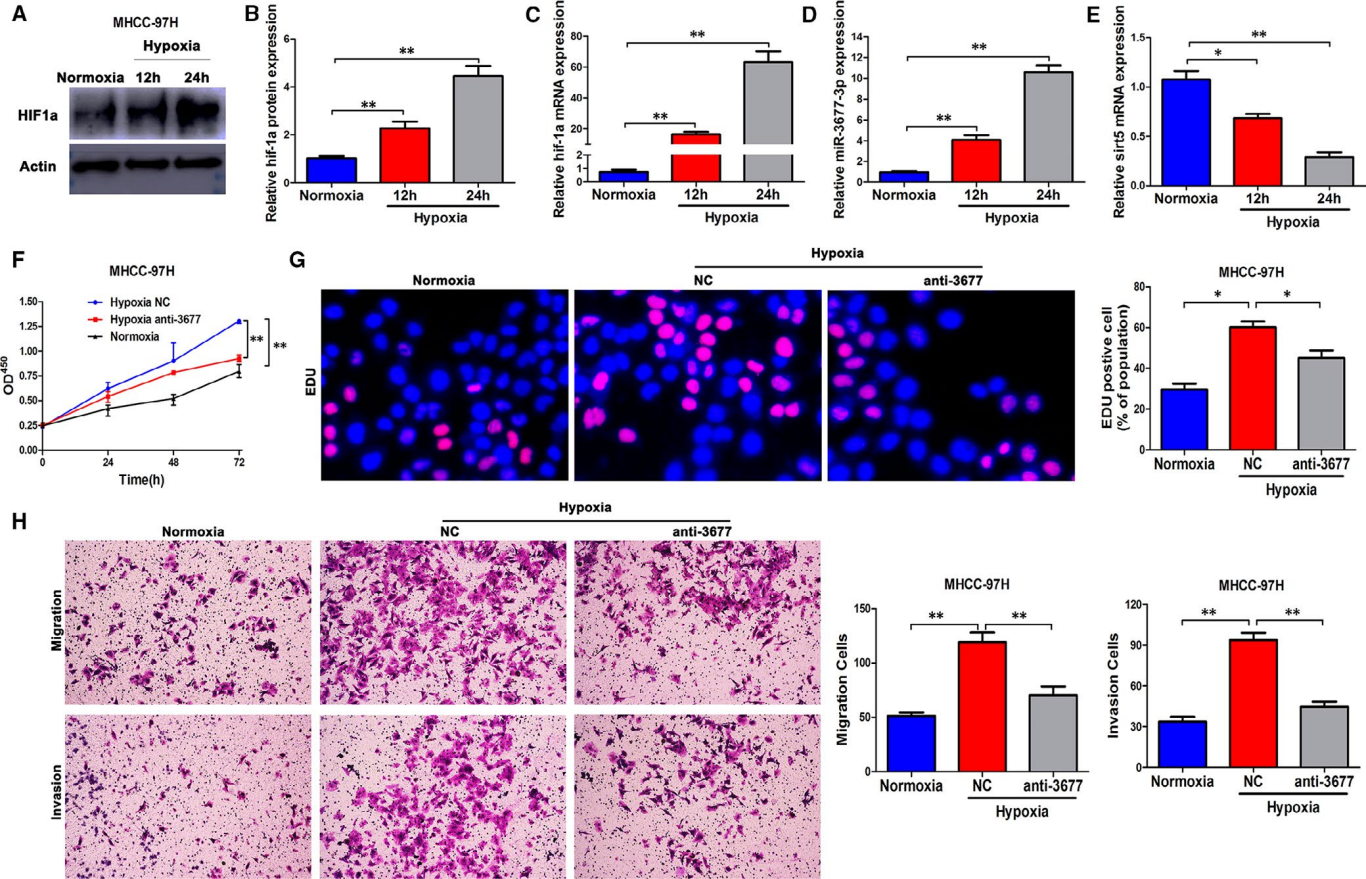
miR‐3677‐3p is up‐regulated by hypoxia and mediates the promoting effects of hypoxia on proliferation, migration and invasion. (A and B) The expression level of HIF‐1α at different time points in a hypoxia condition, beta‐actin was the internal control. C‐E, The expression levels of HIF‐1a, miR‐3677‐3p and SIRT5 in MHCC‐97H cells cultured in normoxia and hypoxia. F, OD450 in CCK‐8 in MHCC‐97H cells in normoxia, and the same cells transfected with miR‐3677‐3p inhibitor in hypoxia. G, EDU percentage of MHCC‐97H cells in normoxia, and the same cells transfected with miR‐3677‐3p inhibitor in hypoxia. H, Transwell assays in MHCC‐97H cells in normoxia, and the same cells transfected with miR‐3677‐3p inhibitor in hypoxia. *P < .05, **P < .01

HCC, one of the hypoxia‐related cancers, exhibits more aggressive features in response to the hypoxia signalling network. Reports of hypoxia and miRNAs in liver cancer are necessary. We detected the expression of all of the genes in the hypoxia‐miR‐3677‐3p‐SIRT5 axis after overexpression of HIF‐1a (Figure 8A,B). The expression of miR‐3677‐3p went up, and SIRT5 decreased in response to hypoxia (Figure 8D,E). Tests of HCC proliferation, migration and invasion showed a powerful tendency towards proliferation and invasion under these conditions (Figure 8F‐H).

Moreover, the TCGA database had the same predicted outcomes about the positive relationship between miR‐3677‐3p and HIF‐1a and negative‐related SIRT5 (Figure S3). Effective tests in HCC grow and transferability when we knocked down the miR‐3677‐3p or overexpressed SIRT5 in hypoxia conditions proved powerful tendency of proliferation and invasion, respectively (Figure 8F,G,H, Figure S4E,F). Therefore, we concluded that hypoxia accelerated HCC progression via the miR‐3677‐3p‐SIRT5 axis.

## 4 DISCUSSION

Large numbers of miRNAs have been reported to modulate the pathological processes of malignancies, such as differentiation, migration and invasion of cancer cells. At the beginning of this study, we noted that there was an apparent abnormal overexpression of miR‐3677‐3p in HCC, while SIRT5 expression was very low in HCC tissues. With the support of databases, we identified a poor prognosis in the high‐miR‐3677‐3p group, which was associated with several malignant characteristics, such as tumour‐node‐metastasis (TNM) stage (III + IV), Edmondson stage (III + IV), venous invasion and multiple tumour nodes. Although there has been no prior research on this miRNA, we could easily explore research into other miRNAs and HCC progression. miR‐21,^18^, ^19^ the miR‐200 family^51^ and some novel miRNAs such as miR‐876 and miR‐1468^23^, ^24^ have been well studied. Moreover, it is known that SIRT5 is down‐regulated in HCC, which could be considered an underlying biomarker for the treatment of HCC.

Sirtuin 5 (SIRT5) was initially identified in 1990s and most researchers believed that since it was localized in the mitochondria, it played significant roles in gene expression and molecular modification regulation. Previous studies have claimed that SIRT5 differential expression could lead to decreased cell growth, endoplasmic reticulum stress (ER stress), senility and apoptosis in various cancers.^40^, ^41^, ^42^, ^44^ However, three studies have indicated that SIRT5 can serve as a promoter of HCC.^46^, ^47^, ^49^ Although these results when weighed against its relatively low‐expression in HCC tissues and cell lines seems to beyond common sense, these findings are contributing to revealing the real function of SIRT5 in liver cancer. It is gratifying that two researchers believed that SIRT5 suppressed the development of HCC. There are still disputes about the function of SIRT5 in HCC. In our study, functional assays have shown that SIRT5 acts as an inhibitor in HCC.

Migration and invasion are the two main aggressive behaviours of any kind of solid tumour, especially HCC. Research into the causes and signalling of these two features are gradually becoming more complete. Increasing numbers of studies have focused on the tumour microenvironment and the cancer‐promoting effect of hypoxia. Hypoxia is a common characteristic of solid tumours and plays an irreplaceable role in tumour heterogeneity, which causes different gene expression and protein secretion patterns in different tumour nodules. A hypoxic environment is closely related to metastasis, apoptosis and invasion of many tumour types, and therefore, identifying pathways and downstream targets of hypoxic conditions might lead to therapeutic candidates for metastatic HCC.

Angiogenesis, reprogramming of metabolism, extracellular matrix remodelling and promotion of inflationary cell chemotaxis have all turned out to be consequences of hypoxia.^9^, ^10^, ^11^, ^12^, ^13^, ^14^ In our study, we found that miR‐3677‐3p was overexpressed in hypoxic conditions, which led to promotion of carcinogenesis. We have delineated the relationship between miR‐3677‐3p and HIF‐1a, but how hypoxia or HIF‐1a regulates the expression of miR‐3677‐3p is still unknown. We are preparing a systemic exploration of how HIF‐1a up‐regulates miR‐3677‐3p. According to the prediction results, the pre‐miR‐3677‐3p sequence is provisional, so we still have no effective knowledge about miR‐3677‐3p's promoter.

SIRT5 is a well‐known gene that leads to histone acetylation and has been reported to be related to hypoxia in some low‐quality reports. However, in two cerebral ischaemia model articles, the author reported that ‘genetic deletion of SIRT5 decreased infarct size, improved neurological function and blunted systemic inflammation following stroke’.^52^, ^53^ Beyond the fact that SIRT5 accelerates the damage of ischaemia, we may make a bold hypothesis that in the hypoxic condition of HCC, there is a failure of SIRT5 to be induced, which facilitates carcinogenesis.

## 5 CONCLUSION

In conclusion, the aforementioned results demonstrated that miR3677‐3p could promote cell division, migration and invasion of HCC through the direct down‐regulation of SIRT5 in a hypoxic condition, which provides novel insights for developing therapeutic approaches to HCC. However, this study remains at the stage before clinical application and the mechanism of hypoxia and whether HIF‐1a regulates miR‐3677‐3p directly are not yet established. Therefore, further studies of direct regulation between HIF‐1a and miRNAs or SIRT5 need to be broadened, and we will expound more upon the function of miR‐3677‐3p in different tumour microenvironments.

## CONFLICT OF INTEREST

The authors confirm that there are no conflicts of interest.

## AUTHOR CONTRIBUTION

**Bowen Yao:** Conceptualization (lead); Data curation (lead); Investigation (lead); Methodology (lead); Project administration (lead); Software (lead); Supervision (lead); Validation (lead); Writing‐original draft (lead); Writing‐review & editing (lead). **Yazhao Li:** Conceptualization (equal); Data curation (equal); Investigation (equal); Methodology (equal); Project administration (equal); Resources (equal); Software (equal); Supervision (lead); Validation (lead); Writing‐original draft (lead); Writing‐review & editing (lead). **Yongshen Niu:** Conceptualization (supporting); Data curation (supporting); Investigation (supporting). **Liang Wang:** Data curation (supporting); Methodology (supporting); Software (supporting). **Tianxiang Chen:** Data curation (supporting); Methodology (supporting). **Cheng Guo:** Conceptualization (equal); Project administration (equal); Resources (equal); Supervision (equal). **Qingguang Liu:** Formal analysis (equal); Project administration (lead); Resources (lead); Supervision (lead).

## Supporting information

Fig S1

Fig S2

Fig S3

Fig S4

## Data Availability

The data that support the findings of this study are available from the corresponding author upon reasonable request.
