# Supplementary figures and images for: Hypoxia‐induced miR‐3677‐3p promotes the proliferation, migration and invasion of hepatocellular carcinoma cells by suppressing SIRT5
Source: J Cell Mol Med. 2020 Jun 28;24(15):8718–31. doi: 10.1111/jcmm.15503 (PMC7412699; doi:10.1111/jcmm.15503)

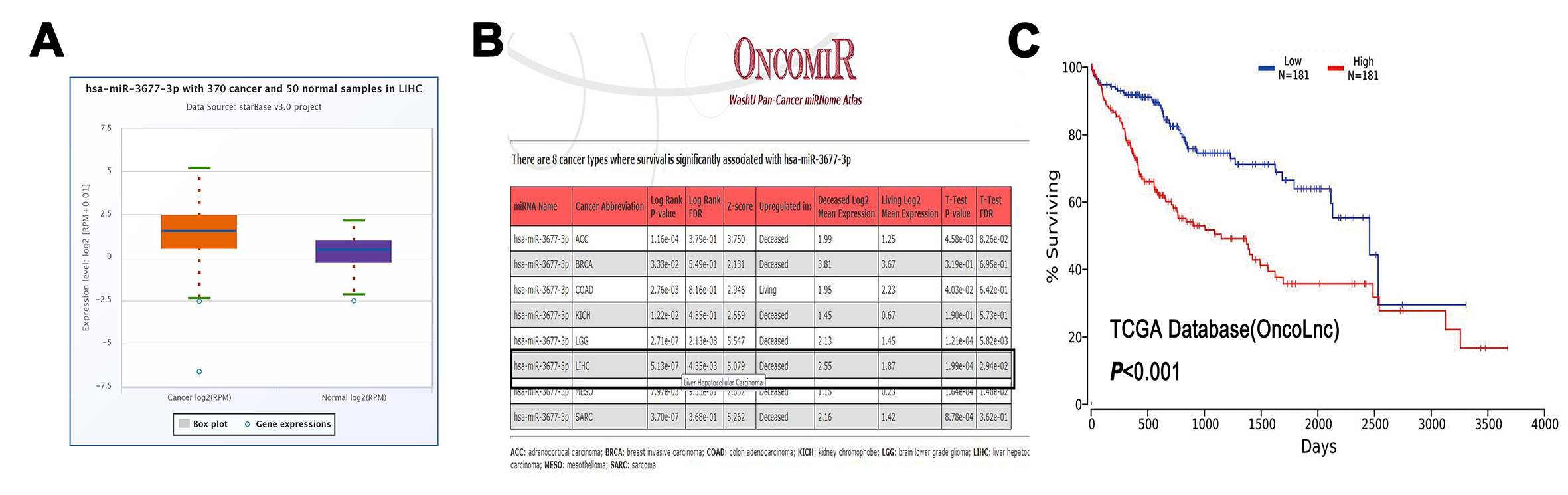

Supplement: Supplementary file 1 — Fig S1 [file JCMM-24-8718-s001.tif]

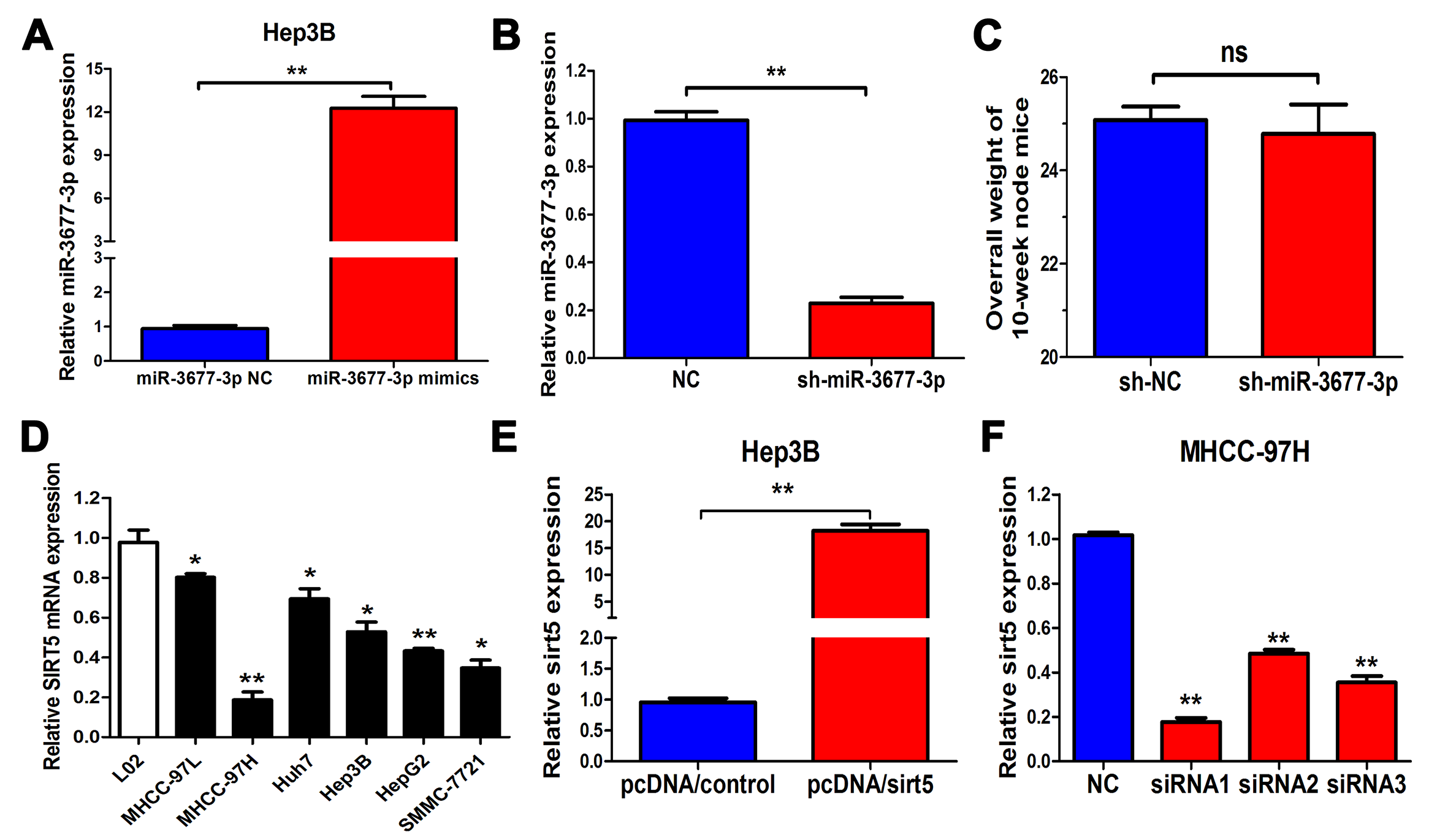

Supplement: Supplementary file 2 — Fig S2 [file JCMM-24-8718-s002.tif]

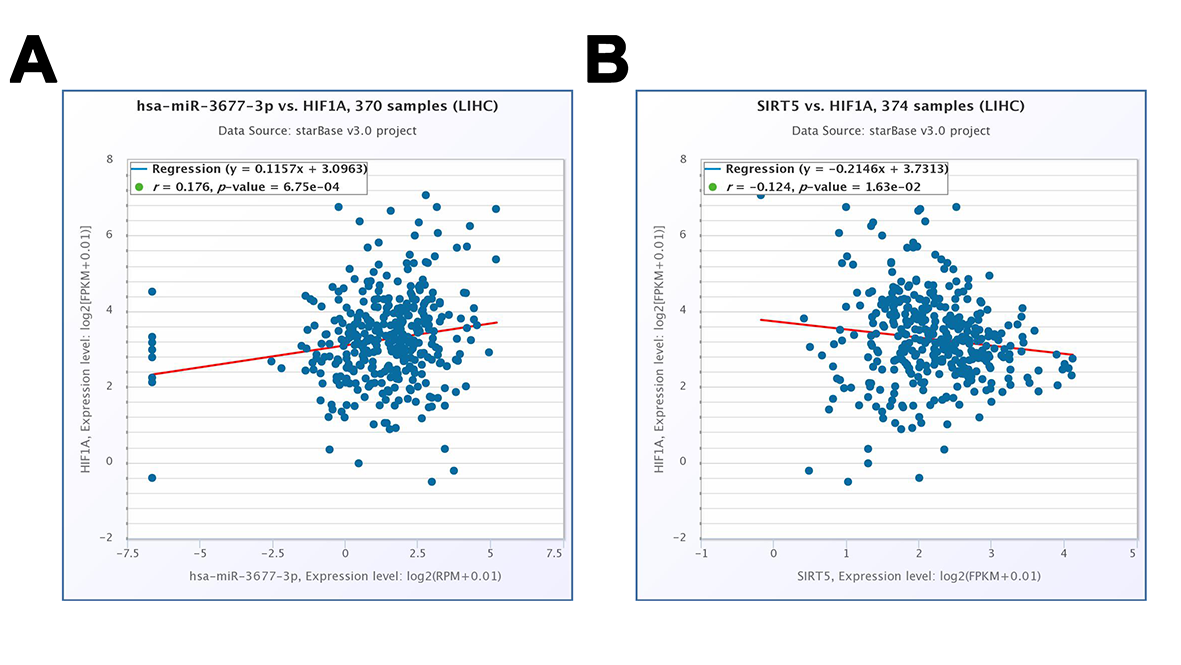

Supplement: Supplementary file 3 — Fig S3 [file JCMM-24-8718-s003.tif]

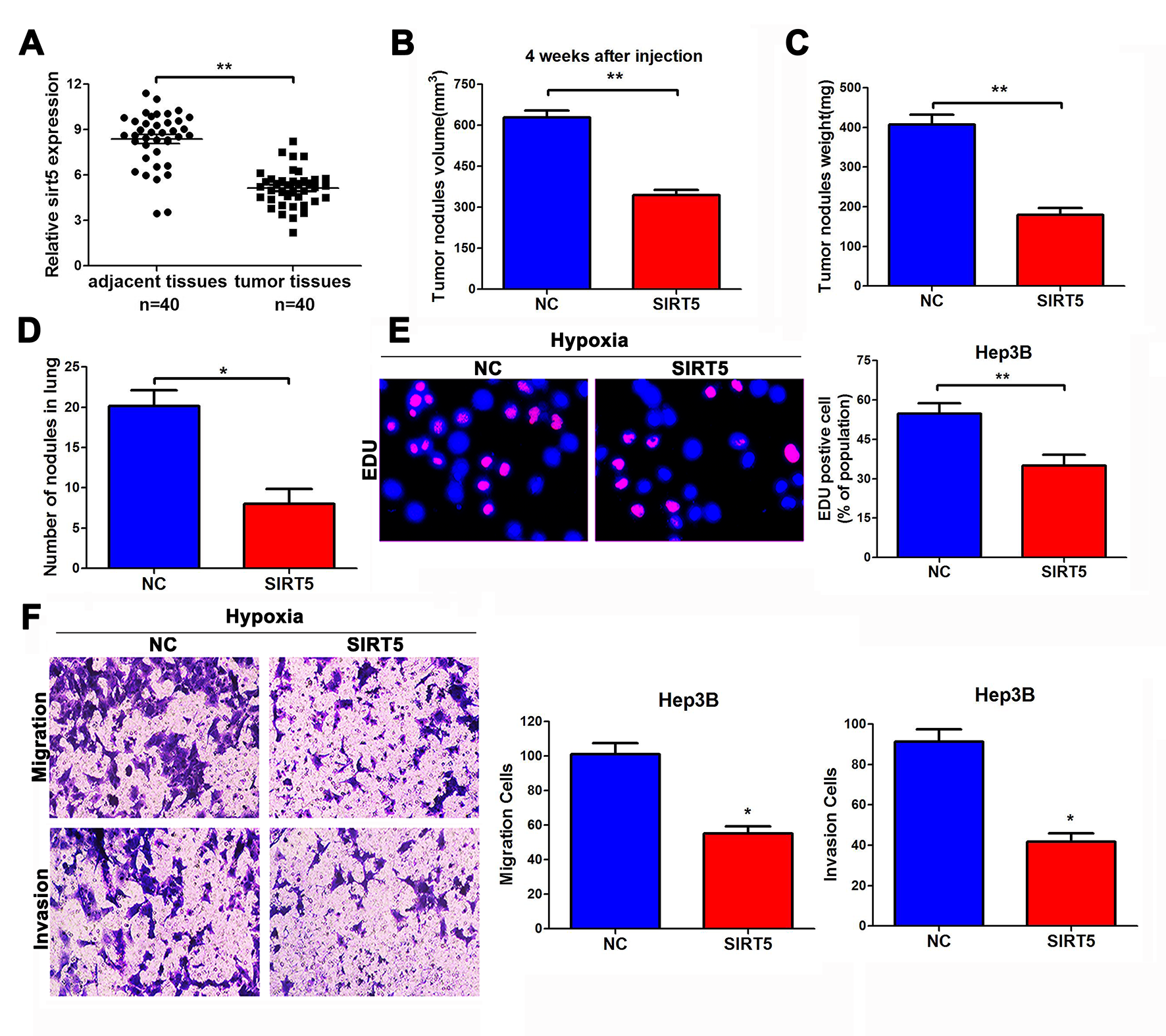

Supplement: Supplementary file 4 — Fig S4 [file JCMM-24-8718-s004.tif]
